# Kisspeptin: A Potential Factor for Unexplained Infertility and Impaired Embryo Implantation

**DOI:** 10.22074/ijfs.2017.4957

**Published:** 2017-02-16

**Authors:** Aaida Mumtaz, Aqsa Khalid, Zehra Jamil, Syeda Sadia Fatima, Sara Arif, Rehana Rehman

**Affiliations:** 1 Medical College, Aga Khan University, Karachi, Pakistan; 2 Biological and Biomedical Sciences, Aga Khan University, Karachi, Pakistan; 3 Civil Hospital, Karachi, Pakistan

**Keywords:** Infertility, Ovarian Stimulation, Intracytoplasmic Sperm Injection, Kisspeptin

## Abstract

**Background:**

Kisspeptin (KP) is a neuropeptide that causes the release of the gonadotropin releasing hormone, which controls hypothalamo pituitary ovarian axis and exerts a number of peripheral effects on reproductive organs. The primary objective of this study was to compare baseline KP levels in females with different types of infertility and identify possible correlations with risk of failure to conceive, preclinical abortion and pregnancy after intracytoplasmic sperm injection (ICSI).

**Materials and Methods:**

A longitudinal cohort study was carried out from August 2014 until May 2015 by recruiting 124 female patients undergoing ICSI, after obtaining ethical approval from the Australian Concept Infertility Medical Center. Cause of infertility due to male, female and unexplained factors was at a frequency of 32 (24%), 33 (31%) and 59 (45%) among the individuals respectively. KP levels were measured by ELISA assay before the initiation of the ICSI treatment protocol. Outcome of ICSI was categorized into three groups of non-pregnant with beta-human chorionic gonadotropin (β-hCG)<5-25 mIU/ml, preclinical abortion with β-hCG>25 mIU/ml and no cardiac activity, and clinical pregnancy declared upon confirmation of cardiac activity. Results based on cause of infertility and outcome groups were analyzed by one-way ANOVA.

**Results:**

Females with unexplained infertility had significantly lower levels of KP when compared with those with male factor infertility (176.69 ± 5.03 vs. 397.6 ± 58.2, P=0.001). Clinical pregnancy was observed in 28 (23%) females of which 17 (71%) had a female cause of infertility. In the non-pregnant group of 66 (53%) females, common cause of infertility was unexplained 56(85%). A weak positive correlation of KP levels with fertilized oocytes and endometrial thickness was observed (P=0.04 and 0.01 respectively).

**Conclusion:**

Deficiency of KP in females with unexplained infertility was associated with reduced chances of implantation after ICSI.

## Introduction

Infertility has been recognized as a disease that requires timely diagnosis, recognition and treatment in terms of assisted reproduction. The prevalence of infertility ranges from 7 to 20% in populations across the globe (1). In 30% of infertile couples, male factors are diagnosed as the cause of infertility (2). Regarding females, structural causes of infertility include tubal damage due to pelvic inflammatory disease, endometriosis and uterine anomalies (3). The dysfunction of the hypothalamic pituitary ovarian axis (HPO) is also thought to be a key player resulting in infertility in many such situations. However, infertility in the presence of normal semen parameters, ovulatory concentration of serum progesterone in mid-luteal phase, tubal patency and a normal uterine cavity is often diagnosed as unexplained infertility (4). Assisted reproductive technology (ART) comprise *in vitro* fertilization (IVF) and intracytoplasmic sperm injection (ICSI) with the latter shown to have a higher success rate (5). With both ARTs, higher failure is observed in couples with unexplained infertility as compared with other infertile subgroups, suggesting a need to explore other key factors that may play a role even in the absence of known abnormalities (6). Furthermore, studies linking unexplained infertility with higher rate of failure to implant after ART highlight the role of interleukins and pro-inflammatory factors in assisting the invasion of the uterine wall (7). In general, the subfertile population is at a greater risk of preclinical abortion. Likewise, its frequency prevails between 30 and 50% of cases who receive ART. Both decreased receptivity of the uterus and chromosomal abnormality in the fetuses are thought to trigger this response, however, other factors that may lead to loss of pregnancy between the time of implantation and onset of menses still remains unknown (8).

Recently, the role of serum kisspeptin (KP) in regulating HPO axis and maturity of oocytes has been aptly investigated (9). KP, an RF-amide peptide coded by the KISS1 gene, was initially believed to be a tumor suppressor gene. The KP receptor, previously known as GPR54, plays an important role in maintaining fertility in both human and other animal species (10). About 40% of gonadotrophin releasing hormone (GnRH) neurons express the GPR54 receptors (11). Neurons located in the hypothalamus secrete KP, thus triggering KP receptors (GPR54) on the GnRH neurons and leading to the secretion of GnRH (9). Besides this, KP is triggered at the onset of puberty and relays information about energy stores of the body to the central nervous system (CNS) by modulating negative and positive feedback of gonadal steroids. Aside from its wide distribution in the brain, KP receptors are highly expressed in the pancreas, placenta, uterus, small intestine, kidney, lung, liver and heart (12). Studies have also suggested other roles for Kisspeptin such as the emergence of KP as a key regulator of the central mammalian reproductive axis along with its role in placentation and pregnancy. This has lead to the exploration of its probable therapeutic role in treating certain forms of infertility (9, 13). We therefore aimed to study the levels of baseline serum KP in infertile females with various types of infertility and examine whether it correlates with the risk of failure to conceive, preclinical abortion and pregnancy after assisted reproduction by ICSI.

## Materials and Methods

A longitudinal cohort study was carried out from August 2014 to May 2015, after Ethical approval was obtained from the Australian Concept Infertility Medical Center. One hundred and twenty-four female patients were recruited after receiving their written informed consent to participate in the study. Females were between 20 and 40 years of age (mean of 32.16 ± 4.8 years), had a mean body mass index (BMI) of 24.19 ± 2.3 kg/m^2^, were segregated into three groups on the basis of their infertility and all were recommended for ICSI. Cause of infertility due to male, female and unexplained factors was at a frequency of 32 (26%), 33 (27%) and 59 (47%) respectively. The first group consisted of those with a male factor infertility (e.g varicocele, prior surgeries and semen abnormalities). The second group comprised females with diagnoses of uterine fibroids (n=2), endometriosis (n=20) and tubal blockade (n=11). The third group comprised 59 patients with unexplained infertility. Females with metabolic disorder (polycystic ovaries) and endocrine disorders such as thyroid dysfunction and abnormal prolactin levels were excluded from the study. Couples diagnosed with both male and female infertility factors were excluded from the study.

Serum samples were collected on the second day of the menstrual cycle before commencement of down-regulation of ovaries. KP was measured using the KISS-1-ELISA Kit (Shanghai, China). The analytical sensitivity of the KP kit was 10.16 ng/L and intra- and inter-assay coefficients of variation was less than 10 and 12% respectively.

### Treatment protocol

Down-regulation of ovaries by 1 mg of subcutaneous buserelin acetate (Suprefact, Sanofi) on day 21 of previous menstrual cycle was followed by controlled ovarian stimulation with Gonal-f (Merck Sereno) from the second day of periods. Confirmation of maturity of follicles to ≥18 mm in diameter was assessed by transvaginal scan (TVS) and cycles were cancelled when follicles failed to develop in response to gonadotropin stimulation. Endometrial thickness was gauged on the day of ovulation induction in the mid sagittal plane by two-dimensional ultrasound with a 7.5-MHz vaginal probe (Hitachi EUB 525, Hitachi, Japan). Oocytes were retrieved from mature follicles (20 mm in diameter), 36 ± 1 hours after injection of human chorionic gonadotropin (hCG, Ovitrelle 250), on the 14^th^, 15^th^ or 16^th^ day of stimulation. Semen samples were retrieved by masturbation and sperms were then immobilized by 7% polyvinyl pyrrolidone after which microinjection was performed (Leica DMIRB, Leica Microsystems, Wetzlar, Germany). Finally, the microinjected oocytes were incubated for 16-18 hours at 37°C, 6% CO2 and 5% O2.

### Outcome measure

Patients were categorized on the basis of β-hCG concentrations on the 14^th^ day after egg collection and on the basis of sonographic evidence of an intrauterine gestational sac 14 days after β-hCG measurement. Females with beta hCG<25 mIU/ml were declared as non-pregnant, pre-clinical abortion was labelled on a beta hCG>25 m IU/ml but with no cardiac activity on ultrasound while clinical pregnancy was confirmed based on higher levels of beta hCG along with intrauterine gestational sac and cardiac activity on TVS. The implantation rate (IR) was calculated as the number of pregnancies per embryo transferred (14), clinical pregnancy (CP) was established by the presence of an intrauterine gestational sac confirmed by TVS per number of patients at the start of cycle (15).

### Statistical analysis

Data were analyzed by SPSS version 21 (IBM statistics, Chicago, IL) and was expressed as mean ± SD/standard error of mean wherever appropriate. Analyses were undertaken for the whole group and independently in sub-groups according to the infertility factor. Variation in KP levels in females with different types of infertility and different outcomes after ICSI was compared by Analysis of variance (ANOVA) and Tukey’s Post-hoc test. Pearson’s correlation coefficient was used to test correlation between KP levels and the study variables. In all cases a P<0.05 was considered significant.

## Results

Table 1 summarises the characteristics of the study population with respect to type of infertility. Significant difference in KP levels amongst these groups was observed (P<0.001). Post-hoc analyses of KP levels revealed significantly reduced levels in female and unexplained infertility factor sub-groups when compared with those with a male factor infertility (P=0.035 and P<0.001, respectively).

**Table 1:** Clinical variables in different types of infertility


Variable	Male infertility	Female infertility	Unexplained infertility
	n= 32	n=33	n=59

Kisspeptin (ng/L)	397.6 ± 58.2^*^^	257.11 ± 24.4^*^	176.69 ± 5.03
No of oocytes fertilized	5.8 ± 0.2	6.02 ± 0.22	5.83 ± 0.16
Endometrial thickness (mm)	8.3 ± 0.6	9.90 ± 3.2^*^	6.74 ± 0.36
No of transferred embryos	1.5 ± 0.1^*^	1.37 ± 0.11^*^	1.83 ± 0.06
Number of gestational sacs	0.3 ± 0.1^^^	0.75 ± 0.13^*^	0.102 ± 0.05
Implantation rate	19.27 ± 6.35	44.9 ± 6.74	5.08 ± 2.88


Blood collected on the second day of cycle before initiation of stimulation. Data expressed as mean ± SD, except kisspeptin levels expressed as mean ± SEM. Results compared by One-Way ANOVA, significant difference (P<0.05) on Post-hoc analysis with unexplained infertility is expressed as * while that between male infertility and female infertility factors is expressed as ^.

**Table 2:** Stratification of outcomes based on type of infertility


Variable	Male infertility			Female infertility			Unexplained infertility		
		Non pregnant n=6	Preclinical abortion n=18	Clinical pregnancy n=8	Non pregnant n=4	Preclinical abortion n=12	Clinical pregnancy n=17	Non pregnant n=56	Preclinical abortion n=0	Clinical pregnancy n=3

Serum KP (ng/L)	352.8 ± 15.9	384.2 ± 38.0	461.1 ± 43.0^*^	163.1 ± 21.5	164.9 ± 2.0	203.3 ± 12.0^*^	171.2 ± 3.6	0 (0%)	278.9 ± 24.9^*^
Oocyte maturation rate	81.8 ± 11.5	93.4 ± 3.7	89.4 ± 5.4	100 ± 0.0	98.4 ± 1.0	97.7 ± 1.0	75.9 ± 1.7	0 (0%)	85.4 ± 0.6
Fertilization rate	63.9 ± 8.7	79.7 ± 2.7	74.1 ± 5.0	82.0 ± 1.6	83.0 ± 1.4	81.3 ± 1.2	59.9 ± 1.4	0 (0%)	76.9 ± 0.5
Implantation rate	0	0	77.0 ± 8.8	0	0	87.2 ± 5.0	0	0 (0%)	3


Data expressed as mean ± SD, except Kisspeptin (KP) levels which are expressed as mean ± SEM. Highest level of KP was observed in clinical pregnancy group in all stratifications (expressed as *).

**Fig.1:**
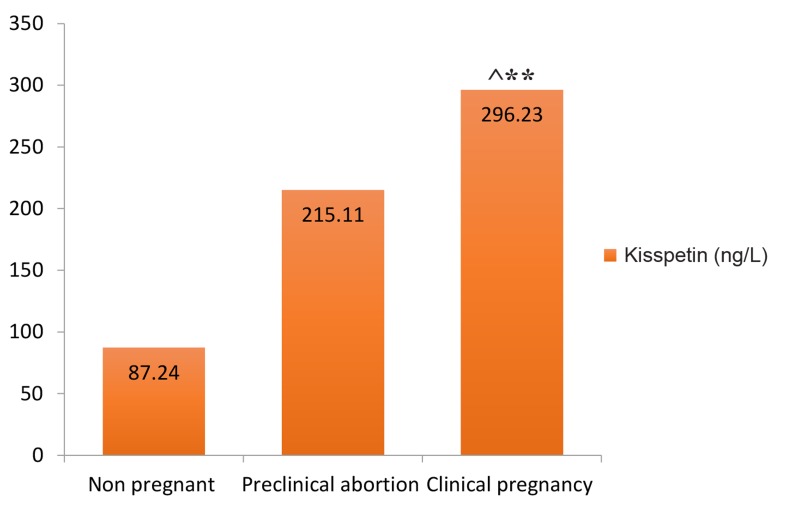
Mean levels of Kisspeptin (ng/L) in groups segregated on the bases of ICSI outcomes. ICSI; Intracytoplasmic sperm injection, **; Clinical pregnancy group versus non-pregnant P<0.001, and ^; Clinical pregnancy versus pre-clinical abortion P<0.05. Error bars represent SEM.

After ICSI, 66 (53%) patients failed to conceive, 30 (24%) reported with preclinical abortions while 28 (23%) were confirmed as clinically pregnant. Mean ± SEM values of KP were 87.24 ± 14.94 ng/L in non-pregnant females and 215.11 ± 34.14 ng/L in pre-clinical abortion while it was 296.23 ± 12 ng/L in patients with clinical pregnancy (Fig .1).

Although variation in KP levels between non-pregnant and pre-clinical abortion groups was marginally significant (P=0.044), it was found to be highly significant between non-pregnant and clinically pregnant females (P<0.001). The outcomes of ICSI treatment based on type of infertility are presented in Table 2. Highest number of clinical pregnancies was achieved in the group of infertile females with diagnosed cause of infertility (51%), however, only 5% of patients with unexplained infertility got pregnant. A weak but significant positive correlation were observed between KP levels and number of fertilized oocytes (r=0.18, P=0.04) and thickness of the endometrium (r=0.27, P=0.01).

## Discussion

With the advancement of ART, couples have been able to conceive, nevertheless, the outcome is associated with a limited success rate (14). The treatment procedures are equally offered to all females suffering from known and unknown factors that disrupt effective functioning of reproductive organs. We observed the lowest levels of KP in females who had unexplained infertility which is consistent with the hypothesis that mutation in the 5ˊ untranslated region (UTR) (deletion, rs5780218) in Exon 1 of KISS1 may disrupt HPO axis, reduce KP levels and likely to be one of the reasons of unexplained infertility (16). Cytokines play an active role in folliculogenesis, ovulation, fertilization and implantation (17). KP is known to work as a pro-inflammatory cytokine alongside tumor necrosis factor α and regulates trophoblast cell invasion, leading to implantation (18, 19). Many studies have linked unexplained infertility with low implantation rates while highlighting the role of interleukins and pro-inflammatory factors in helping the invasion of the uterine wall (7). In our study, reduced KP levels in females with unexplained infertility was associated with inadequate egg maturation, reduced fertilization, thinner endometrial lining and failure of implantation of the blastocyst. This is probably the basis by which injection of a single dose of KP-54 triggers egg maturation, fertilization and implantation of blastocyst in infertility treatment procedures (20, 21).

Fayazi et al. (22) discovered the presence of a KP/KISS1R signaling pathway in the uterus of 4-day pregnant mouse with a slight expression of KP signaling in the uterus of non-pregnant mice. KP expression thus has a maintenance basal level in peripheral reproductive organs, which is up-regulated at the initiation of pregnancy. A low level of KP in non-pregnant females in our study emphasizes its role in placentation and implantation. This observation is in accordance with the researchers previous work with the difference that KP was estimated after the suppression of HPO axis (23). Preclinical abortion after ICSI is subject to a number of factors including the etiology of infertility (8). The preclinical abortion was observed in 24% of the study population, which mainly comprised females who had uterine fibroids, endometriosis and tubal blockade. These results are contradictory to those in studies that observed similar pregnancy loss with different types of infertility (24). We nonetheless observed that women with preclinical abortions had a higher KP level than patients who failed to conceive. This may be explained by the existence of a circadian KP expression in a full-term human placenta of healthy women that regulates trophoblastic invasion and probably pregnancy maintenance (25).

During the menstrual cycle, secretory changes in the endometrium account for coordinated signal exchange between hormonally primed endometrium and functional embryo for the implantation of embryo. This is made possible by the interplay of hormones and cytokines, and studies have demonstrated that increased endometrial thickness is associated with higher pregnancy rates (26). A cut-off value of 8mm thickness was considered to be optimal for embryo implantation after ICSI in the female population of Pakistan (17). We observed that a thicker endometrial lining was associated with a higher implantation rate when compared with females who could not conceive at all. Moreover, thickness of the endometrium was at its lowest in patients with unexplained infertility, who also had minimum levels of serum KP.

The prevalence of infertile couples with unexplained factors is estimated at 30% (27). To the best of our knowledge, this is the first study that supports association of reduced KP in unexplained infertile females with reduced fertilization of oocytes, implantation of blastocyst and hence conception. Improving the success rate after IVF and ICSI for couples with unexplained infertility is thus what further work should be aimed at. This study is uni-centric, has a small sample size and included infertile couples with both male and female factors, which all may bias the observed correlation between KP levels and unexplained infertility. In addition, we did not stratify the male infertility factor based on sperm parameters including count, motility and morphology. This may be a confounding factor for low implantation rate in females with diagnosed male infertility factor.

## Conclusion

Low level of KP in females with unexplained infertility before the initiation of the ICSI protocol was seen. One of the factors causing unexplained infertility may thus be low KP levels. The level of KP has an impact on fertilization of oocytes, preparation of endometrial beds for implantation of embryo and hence successful pregnancy after ICSI. This observation urges the need of further studies at genetic and molecular levels to define and explain the role of KP for preservation of conception and continuation of pregnancy in females with unexplained infertility.
